# Dilated cardiomyopathy as the initial presentation of Becker muscular dystrophy: a systematic review of published cases

**DOI:** 10.1186/s13023-022-02346-1

**Published:** 2022-05-12

**Authors:** Gaspar Del Rio-Pertuz, Cristina Morataya, Kanak Parmar, Sarah Dubay, Erwin Argueta-Sosa

**Affiliations:** 1 Department of Internal Medicine, Texas Tech University Health Sciences Center, 3601 4th St, Lubbock, TX 79430 USA; 2 Department of Clinical and Diagnostic Sciences, University of Alabama at Birmingham, Birmingham, AL 35294 USA; 3 Division of Cardiology, Texas Tech University Health Sciences Center, Lubbock, TX 79430 USA

**Keywords:** Dilated cardiomyopathy, Becker muscular dystrophy, Heart failure, Systematic review

## Abstract

There are scarce publications regarding the presentation and outcome of Becker muscular dystrophy in adulthood when idiopathic dilated cardiomyopathy is the initial disease manifestation. We performed a systematic review using Medline, Embase, Cochrane, and Scopus to identify cases of adults with idiopathic dilated cardiomyopathy who were subsequently diagnosed with Becker muscular dystrophy from inception through August 2020. Six cases were found. We identified young males (Median age: 26 years) with Becker muscular dystrophy who first presented with dilated cardiomyopathy. Most patients initially presented with congestive heart failure symptoms (5/6, 83%), and had a median left ventricular ejection fraction of 23%. One case did have calf pseudohypertrophy. Musculoskeletal symptoms later appeared one to six years after the initial dilated cardiomyopathy presentation. Heart transplantation was the most common management strategy (4/6, 67%). A left ventricular assist device was used in one case as a bridge to heart transplant. Dilated cardiomyopathy can be the initial presentation of Becker muscular dystrophy in the third to fourth decades of life in adult patients, and musculoskeletal symptoms can be subclinical.

## Introduction

Dystrophinopathies are X-linked recessive muscle diseases caused by a mutation in the Duchene Muscular Dystrophy (DMD) gene. It encodes the sarcolemma protein, dystrophin, that is present in skeletal and cardiac muscle [1]. Dystrophinopathies have a spectrum of phenotypes. The most severe disease, DMD, is characterized by the early onset of rapid progressive disease in childhood. Becker muscular dystrophy (BMD) is a milder disease variant; it is a less common form and has a later onset compared to DMD. Lastly, X-linked dilated cardiomyopathy, is another phenotype in which the skeletal muscle can be clinically spared.

It has been classically described that the onset of dystrophinopathies occurs during childhood, initially presenting with musculoskeletal involvement and subsequently the development of myocardial dysfunction in late childhood or early adulthood [2]. Depending on the phenotype, the amount of myocardial involvement in X-linked muscular dystrophies varies. For example, myocardial dysfunction can be subtle in wheelchair-bound patients since there is decreased demand of the heart (i.e. DMD), or the dysfunction can be more obvious in patients that are ambulatory and present as a dilated cardiomyopathy (DCM) syndrome (i.e. BMD) [3]. Except for X-linked DCM, DCM is an uncommon initial presentation for most X linked muscular dystrophies.

When an adult presents with an unknown DCM etiology, the spectrum of dystrophinopathies is often not included in differential diagnosis because patients may not have had musculoskeletal symptoms in the past. The amount of publications regarding the presentation and outcome of either BMD or DMD in adulthood is scarce when idiopathic DCM is the initial manifestation of the disease. The present systematic review aims to describe and consolidate the most current available evidence in which adult patients with unknown dystrophinopathy had idiopathic DCM before the onset of musculoskeletal symptoms.

## Methods

A comprehensive search of several databases from each database's inception to August 4th, 2020. The databases included Ovid MEDLINE(R) and Epub Ahead of Print, In-Process & Other Non-Indexed Citations, and Daily, Ovid EMBASE, Ovid Cochrane Central Register of Controlled Trials, Ovid Cochrane Database of Systematic Reviews, and Scopus. The search strategy was designed and conducted by an experienced librarian with input from the study's principal investigator using a combination and variation of the terms ‘’Becker muscular dystrophy’’, ‘’Duchene muscular dystrophy’’, ‘’X-linked dilated cardiomyopathy’’, ‘’ dystrophinopathy’’, ‘’cardiomyopathy’’ and ‘’adult’’. Controlled vocabulary supplemented with keywords was used to search for cardiomyopathy and dystrophinopathy in adults. The actual strategy listing all search terms used and how they are combined is available in the Appendix. This study has been registered at PROSPERO International prospective register of systematic reviews under registration no. CRD42020203663.

Titles and abstracts of all articles retrieved using the search strategy were initially screened, reviewed, and verified independently by two authors GDRP and CM, with disagreements mediated through discussion with a third review author EAS. The full texts of potentially eligible articles were reviewed by GDRP and CM, with disagreements mediated by EAS. Selection criteria used to identify studies included: human studies in either English or Spanish, clinical trials, prospective or retrospective observational studies, case reports, and case series where adult patients (older than 17 of age) with unknown dystrophinopathy presented with idiopathic DCM before the onset of musculoskeletal symptoms. Studies excluded were case-patients younger than 18 years of age and/or had a known BMD or DMD or any musculoskeletal symptoms before the onset of DCM.

For all included articles, information was extracted independently by two authors (GDRP and CM) into a standardized form for assessment of study quality and evidence synthesis, any discrepancy was mediated by a discussion with a third author (EAS). The information extracted from each article, if available, was: Main author, published date, study type, and the number of patients reported. From each patient-reported, we extracted age, gender, race, chief complaint at admission, creatine phosphokinase at admission, presence of calf pseudohypertrophy during the initial presentation, ejection fraction at admission, NYHA stage at admission, gene deletion, presence of a family history of dystrophinopathies, musculoskeletal symptoms described at presentation, age of initial musculoskeletal symptom onset, management received, results of Cardiovascular magnetic resonance imaging (CMR), and skeletal and cardiac muscle biopsy. In the case of missing or unclear data, corresponding authors were contacted to provide further information.

The quality of each study was evaluated using the CARE Checklist. The previous is a 13-item checklist that provides a framework to satisfy the need for completeness and transparency for published case reports [4]. Each case report was evaluated independently by two coauthors (GDRP and CM) and disagreements mediated by consensus with a third author (EAS).

## Results

**Fig. 1:**
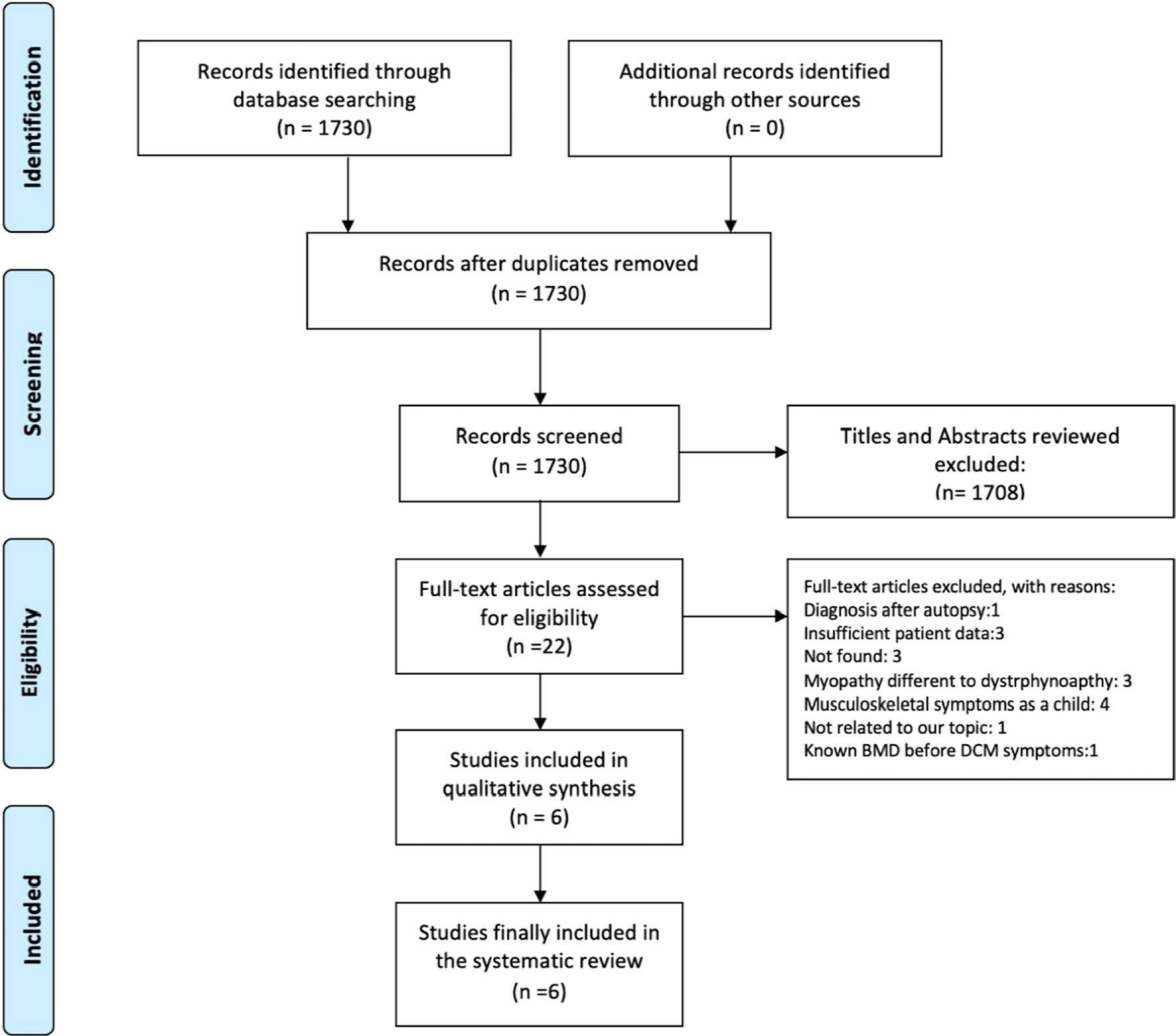
Prisma flow chart

After reviewing 1730 abstracts, 22 articles were considered potentially eligible and evaluated in-depth. After full-text review, 16 articles were later rejected. Five full manuscripts and one poster presentation were included in the present systematic review. Figure 1 summarizes the literature review process. All studies included were case reports. Table 1 shows the demographics, clinical characteristics, diagnostic tests, management, and outcome for each of the six patients that were totally included [5–10].

**Table 1:** Clinical Characteristics of case reports included

	Papa et al. [5]	Bojaras et al. [6]	Finsterer et al. [7]	Guo et al. [8]	Juan-Mateu et al. [9]	Piccolo et al. [10]
Age of presentation	27	22	26	23	26	31
Gender	Male	Male	Male	Male	Male	Male
Initial complaint	NA	SOB and Orthopnea	NA	Progressive fatigue and exertional dyspnea	Asymptomatic*	SOB
Initial diagnosis	DCM	DCM	DCM	DCM	DCM*	DCM
Left ventricular EF at presentation	#	25%	#	35%	20%	17%
CK level at initial presentation (IU/l)	#	1964	#	4688	1400	#
Calf pseudohypertrophy at initial presentation	No	No	No	Yes	No	No
Management	Heart transplant performed 2 years after diagnosis	LVAD—Heart Transplant 3 years after diagnosis	Heart transplant—1 year after diagnosis	Not specified	Medical management	Heart transplant right after presentation
Age of initial musculoskeletal symptoms	31	23	33	No symptoms described	No symptoms described	No symptoms described
Musculoskeletal complaint	Lower limb weakness	Lower limb weakness	Lower limb weakness and thigh wasting	No symptoms described	No symptoms described	No symptoms described
BMD gene abnormality	BMD deletion exons 3–4	BMD deletion	BMD deletion exons 48 and 49 and the intronic short tandem repeat sequence STR 49	BMD deletion of exon 11–12	BMD Mosaicism Exon 44	BMD deletion exon 45–47
Family history of BMD	No	Brother	No	No	No	No
Confirmed family history of muscular dystrophy	No	No	No	No	No	Two uncles
CMR Findings	#	DCM with severe mitral valve regurgitation	#	LV lateral wall subepicardial replacement with fibrotic tissue and fat	#	#
Skeletal muscle biopsy	#	#	Dystrophic changes	#	Dystrophic changes	Dystrophic changes
Endomyocardial biopsy	#	Non-specific changes of DCM	#	#	#	Dystrophic changes
Outcome—Follow up	Death—21 years after presentation	Death—3–4 years after presentation	Alive—8 years after presentation	Unknown	Alive 3 years after presentation	Alive 4 years after presentation

^#^Results were not described in the study; *Without symptoms, dilated cardiomyopathy was found incidentally during work up and the incidental finding of an elevated creatinine kinase level

*DCM* Dilated cardiomyopathy, *NA* No available, *SOB* Shortness of breath, *EF* Ejection fraction, none: not reported by the study, *CK* Creatine Kinase, *LVAD* Left ventricular assisted device, *BMD* Becker muscular dystrophy, *CMR* Cardiac magnetic resonance, *LV* Left Ventricle

As described in Table 1, DCM was the initial presentation of BMD identified in young males with a median age of 26. Most patients presented with congestive heart failure symptoms (5/6, 83%) with a median left ventricular ejection fraction (LVEF) of 23%. The median serum creatine kinase (CK) level at admission was 1964 (IU/L). No patients described having musculoskeletal symptoms initially, but one had calf pseudohypertrophy. Heart transplantation was the most common management strategy (4/6, 67%). A left ventricular assist device was used in one case as a bridge to heart transplant. Lower limb weakness was the most common musculoskeletal complaint (3/6, 50%), followed by thigh wasting (1/6, 17%). All of these symptoms appeared one to six years after DCM onset.

The patients included had different loci for the dystrophin gene deletion, ranging from exon 3 to 47. Two patients had a history of muscular dystrophy in the family, and one had a sibling with confirmed BMD. Cardiac magnetic resonance (CMR) was not commonly performed (2/6, 33%), but when obtained, findings included severe mitral valve regurgitation and subepicardial replacement with fibrotic tissue and fat in the left ventricular lateral wall. Skeletal muscle biopsy was performed in half of the cases, and these results described characteristic dystrophic changes. Endomyocardial biopsy was done in less than half of the patients (2/6 33%), in which dystrophic and non-specific DCM changes were found. The longest reported survival was 21 years after the initial presentation.

## Discussion

Becker muscular dystrophy and DMD are a subgroup of X-linked recessive neuromuscular disorders called dystrophinopathies that arise from mutations in the dystrophin gene. Both diseases are characterized by mechanical weakness in skeletal and cardiac myocytes. While patients with DMD have a complete absence of dystrophin and typically present in childhood, BMD is characterized by in-frame mutations in the DMD gene leading to reduced dystrophin protein expression and relatively delayed disease progression [1].

There is a significant variation in the age of onset for BMD, ranging from two to 20 years [11]. Muscular cramps with strenuous physical activity and a delayed ability to jump and run are the initial disease complaints. Muscular weakness classically starts affecting proximal before distal limbs and lower limbs before upper limbs. Cardiomyopathies appear years after the onset of musculoskeletal symptoms [12]. Interestingly, in patients that had DCM as their initial disease presentation, the median age of onset was 20 years, and the musculoskeletal symptoms appeared one to six years after DCM onset.

Dilated cardiomyopathy, followed by arrhythmias, is the most common cardiac abnormality described and the leading cause of death in patients with BMD [13–15]. Dilated cardiomyopathy is present in more than 70% of the population with BMD and typically presents in the third to fourth decade of life with typical heart failure symptoms [16, 17]. Previous studies have shown that there is no correlation between the extent of cardiac and skeletal muscle disease in patients with BMD. Nevertheless, the deletion location has been correlated with the DCM age of onset. For instance, the locus of the dystrophin deletion in one of the patients included in this systematic review (EXON 3–4) has been associated with an early DCM onset, even in patients without an obvious decline in muscle function [18].

Based on these results, DCM can be an initial manifestation of BMD. We consider that this presentation in patients with BMD is possible with little to no musculoskeletal involvement when patients are still able to perform strenuous exercise. The associated mechanical stress on the heart during exercise due to pressure and volume overload could hypothetically be harmful to dystrophin-deficient myocardial cells, producing a continuous damage and repair cycle which lead to myocardial fibrosis and ventricular dilation [19–21].

Serum CK levels are elevated in children with DMD or BMD before the presence of any muscular disease. Skeletal muscle isoenzyme of CK, CK-MM, is usually used as a screening marker to identify if newborns have suspected myopathic disease. In cases where symptoms of BMD or DCM started at a young age, the CK levels eventually reached the normal range in adulthood, as more and more muscles were replaced by fat and fibrosis [22, 23]. Bojoras et al. [6], Guo et al. [8], and Juan-Mateu et al. [9], were the only cases who described levels of serum CK at presentation, and all of them were above normal limits. We, therefore, hypothesize that because these patients did not have any musculoskeletal symptoms, they persistently had elevated serum CK in adulthood, because of active sarcolemma rupture in the muscles that had not been completely replaced by fatty and fibrotic tissue. To our knowledge, it is reasonable to measure CK levels to screen for myopathies, however there is no concise data regarding the sensitivity and specificity of elevated serum CK levels as a screening tool for BMD in adults who present with idiopathic DCM, and more studies are needed to clarify its utility.

In up to 50% of cases with DCM, the exact cause remains initially unknown; this condition is called idiopathic DCM [24]. It is reasonable to include BMD in the differential diagnosis when we approach a young patient with idiopathic DCM, especially when any of the following are present: elevated CK at presentation, calf pseudohypertrophy, and/or musculoskeletal complaints.

Regarding management of BMD-associated DCM, obtaining an ECG and echocardiogram are recommended at the time of BMD diagnosis and every five years thereafter. Cardiac magnetic resonance is gaining acceptance as a more sensitive modality than echocardiography for detecting early regional myocardial fibrosis in BMD. Some groups have recommended a CMR be completed every two years. Previous CMR studies have demonstrated subepicardial gadolinium enhancement in BMD and DMD [1], which was also described in one of our included cases.

There are no specific guidelines for pharmacotherapy in patients with BMD-associated DCM, though guideline-directed medical therapy should be initiated if the LVEF is reduced. Most of the current studies are from non adults with DMD-associated DCM [25]. Some groups have suggested that earlier initiation of beta blockers and angiotensin converting enzyme inhibitors (ACEIs) in patients with myocardial dysfunction secondary to dystrophinopathies may delay the progression of cardiac dysfunction before LVEF is reduced [26, 27]. Although some DMD patient studies indicate that beta blockers preserve cardiac function and survival beyond the effects of ACEIs alone, a recent trial revealed no difference between treatment groups receiving ACEIs alone versus ACEIs with beta blockers when the ACEI dose was adjusted according to the severity of cardiac dysfunction [28–30]. Additionally, eplerenone has been demonstrated to attenuate left ventricular systolic function decline in DMD patients with preserved LVEF and evidence of myocardial disease by CMR [25]. Glucocorticoids are indicated in patients with DMD with declining motor function, and observational studies have shown a potential role for steroids in preserving cardiac function in these patients [31]. However, no prospective trials have been completed, and steroids are not currently indicated in the setting of isolated dystrophin-deficient cardiomyopathy.

Strategies for dystrophin repair include nonsense readthrough therapy, vector‐mediated gene delivery, and exon skipping with synthetic antisense oligonucleotides or genome editing. However, many of these therapies are still in preclinical development, or have not yet been shown to benefit cardiac muscle in DMD patients [32]. Eiliprisen, a phosphonodiamidite morpholino oligomer, induces the skipping of DMD exon 51. Eiliprisen received approval by the FDA in 2016 for DMD treatment, but no evidence exists about the significant benefits specifically on the heart [33]. CRISPR-Cas9 is a new strategy for correcting genetics, studies have demonstrated significant restoration of cardiac dystrophin expression and improvement in cardiac pathology in different preclinical models of DMD [34, 35]. Furthermore, the correction has shown to persist long-term in mice, reinforcing the concept of permanent gene repair with this genomic editing strategy [36]. Novel non-genetic therapies have been studied, such as the use of myostatin inhibitors. Loss of myostatin has been shown to cause increase in skeletal muscle size and improve skeletal muscle function and fibrosis in murine models of DMD, but no effect has been shown in cardiac muscle growth or fibrosis.

Previously, orthotopic heart transplantation was relatively contraindicated in patients with inherited myopathies due to concerns of severe musculoskeletal weakness limiting rehabilitation potential and respiratory muscle dysfunction limiting the ability to wean off of mechanical ventilation postoperatively [16], Despite this Wu et al. [37], demonstrated similar outcomes in terms of survival, cardiac rejection, and transplant vasculopathy between patients with muscular dystrophy and idiopathic DCM. It is important to note that these findings occurred in patients with mild muscular disability and no respiratory muscle involvement. Interestingly, in patients with early DCM onset, the musculoskeletal complaints can start from one to six years after. This means that early heart transplantation could potentially help outcomes in these patients [5]. Papa et al. [5], Bojaras et al. [6], Finsterer et al. [7] and Piccolo et all [10], performed heart transplant in their patients, demonstrating that it is a common and useful management approach. Furthermore, they showed that survival after transplantation may range from one to eight years after orthotopic heart transplant.

The main strength of this study is that it is the first systematic review that consolidates the data available over this specific group of patients who have BMD and initially present with DCM. This study has several limitations. First, the number of patients included in this study was small. This may be attributed to the specificity of this rare disease presentation. Second, all the studies in the literature were case reports. Third, there is a possibility that early symptoms were initially missed or ignored by doctors and/or patients, further delaying the diagnosis of the disease and causing it to present in adulthood instead of childhood. Young people regularly do not complain of symptoms, and the progression of myocardial dysfunction can be transient and gradual, causing a slow decrease in the LVEF that can go unnoticed by the patients due to body compensation to the metabolic requirements. However, there comes a point where the DCM is too severe that the body is unable to compensate for its metabolic demands, causing intolerable symptoms of heart failure. Fourth, the DCM disease course of the patient before they saw a doctor for the first time was not included in any of the cases described. Finally, the patient information described on each case report was scant, for instance, to tackle these limitations authors were contacted for more information but no responses were finally received. This may mean that our article may not be comprehensive enough.

## Conclusions

Dilated cardiomyopathy can be the initial presentation of BMD in adult patients in their third to fourth decades of life before developing musculoskeletal symptoms. Most importantly, dystrophinopathies should be included in the differential diagnosis of a young adult male who presents with DCM and elevated CK levels.

## Data Availability

Data sharing not applicable to this article as no datasets were generated or analyzed during the current study.

## Appendix: Search strategy

### Ovid

Database(s): EBM Reviews—Cochrane Central Register of Controlled Trials June 2020, EBM Reviews—Cochrane Database of Systematic Reviews 2005 to July 31, 2020, Embase 1974 to 2020 July 31, Ovid MEDLINE(R) and Epub Ahead of Print, In-Process & Other Non-Indexed Citations and Daily 1946 to July 31, 2020.

Search Strategy:

**Table:**

**#**	Searches	Results
1	exp Muscular Dystrophy, Duchenne/	21866
2	exp Becker muscular dystrophy/	8682
3	exp dystrophinopathy/	587
4	(((Duchenne or Becker) adj3 carrier*) or "becker dystroph*" or "becker muscular dystroph*" or "becker syndrome*" or "becker type muscular dystroph*" or "beckers dystroph*" or "beckers muscular dystroph*" or "beckers syndrome*" or "beckers type muscular dystroph*" or "beckers-type progressive muscular dystroph*" or "becker-type progressive muscular dystroph*" or BMD or "childhood pseudohypertrophic muscular dystroph*" or DMD or "duchenne dystroph*" or "duchenne muscular dystroph*" or "duchenne syndrome" or "duchenne type muscular dystroph*" or "duchennes dystroph*" or "duchennes muscular dystroph*" or "duchennes syndrome" or "duchennes type muscular dystroph*" or "duchennes-type progressive muscular dystroph*" or "duchenne-type progressive muscular dystroph*" or dystrophinopath* or "morbus duchenne*" or "pseudo hypertrophic myopathic progressive muscular dystroph*" or "pseudohypertrophic childhood muscular dystroph*" or "pseudohypertrophic muscular dystroph*" or "X linked dilated cardiomyopath*" or "XL-dCM").ti,ab,hw,kw	119809
5	1 or 2 or 3 or 4	120433
6	exp Cardiomyopathies/	236580
7	(("glycogen storage disease*" adj5 (heart or cardiac* or myocard*)) or adhalinopath* or "alpha sarcoglycanopath*" or "alpha-sarcoglycanopath*" or "antopol disease*" or "arrhythmogenic right ventricular dysplasia" or "asymmetric septal hypertrophy" or "Barth syndrome" or cardiomyopath* or "cardioskeletal myopathy with neutropaenia and abnormal mitochondria" or "cardioskeletal myopathy with neutropenia and abnormal mitochondria" or "cardiovascular trypanosomiasis" or carditis or "chagas myocarditis" or "chronic progressive external ophthalmoplegia with myopath*" or "congestive heart disease*" or "cpeo with myopath*" or "cpeo with ragged red fiber*" or "danon disease*" or "diabetic cardiomyopath*" or "endocardial fibroelastos*" or "endomyocardial fibroelastos*" or "endomyocardial fibros*" or "familial ventricular hypertrophy" or "glycogen storage disease iib" or "glycogen storage disease type 2b" or "glycogen storage disease type iib" or "heart amyloidos*" or "heart myopath*" or "heart right ventricle dysplasia*" or "hereditary ventricular hypertroph*" or "isolated left ventricular noncompaction" or "isolated noncompaction of the ventricular myocardium" or "isolated non-compaction of the ventricular myocardium" or "isolated noncompaction of ventricular myocardium" or "isolated ventricular noncompaction" or "kearn sayre mitochondrial cytopath*" or "kearn syndrome" or "kearns sayre shy daroff syndrome" or "Kearns Sayre syndrome" or "kearns syndrome" or "kearn-sayre mitochondrial cytopath*" or "kearns-sayre mitochondrial cytopath*" or "kearns-sayre syndrome" or "kearns-sayre-shy-daroff syndrome" or "keshan disease*" or "left ventricular noncompaction" or "left ventricular non-compaction" or "lysosomal glycogen storage disease with normal acid maltase" or "lysosomal glycogen storage disease without acid maltase deficiency" or "muscular dystrophy limb girdle with alpha sarcoglycan deficiency" or "muscular dystrophy limb-girdle with alpha-sarcoglycan deficiency" or "myocardial disease*" or "myocardial ischemic reperfusion injur*" or "myocardial reperfusion injur*" or myocardiopath* or myocarditides or myocarditis or "noncompaction of the ventricular myocardium" or "noncompaction of ventricular myocardium" or "nonobstructive hypertrophic myocardiopath*" or "obstructive asymmetric septal hypertrophy" or "oculocraniosomatic syndrome*" or "ophthalmoplegia plus syndrome*" or "primary adhalinopath*" or "primary myocardial disease*" or "pseudoglycogenosis 2" or "pseudoglycogenosis 2 s" or "pseudoglycogenosis ii" or "pseudoglycogenosis iis" or sarcoglycanopat* or "secondary myocardial disease*" or "ventricular noncompaction" or "ventricular non-compaction" or "X linked cardioskeletal myopathy and neutropaenia" or "X linked cardioskeletal myopathy and neutropenia").ti,ab,hw,kw	337659
8	6 or 7	337659
9	5 and 8	3471
10	limit 9 to ("all adult (19 plus years)" or "young adult (19 to 24 years)" or "adult (19 to 44 years)" or "young adult and adult (19–24 and 19–44)" or "middle age (45 to 64 years)" or "middle aged (45 plus years)" or "all aged (65 and over)" or "aged (80 and over)") [Limit not valid in CCTR,CDSR,Embase; records were retained]	2738
11	limit 10 to (adult < 18 to 64 years > or aged < 65 + years >) [Limit not valid in CCTR,CDSR,Ovid MEDLINE(R),Ovid MEDLINE(R) Daily Update,Ovid MEDLINE(R) In-Process,Ovid MEDLINE(R) Publisher; records were retained]	1171
12	limit 9 to ("all infant (birth to 23 months)" or "all child (0 to 18 years)" or "newborn infant (birth to 1 month)" or "infant (1 to 23 months)" or "preschool child (2 to 5 years)" or "child (6 to 12 years)" or "adolescent (13 to 18 years)") [Limit not valid in CCTR,CDSR,Embase; records were retained]	2735
13	limit 12 to (embryo or infant or child or preschool child < 1 to 6 years > or school child < 7 to 12 years > or adolescent < 13 to 17 years >) [Limit not valid in CCTR,CDSR,Ovid MEDLINE(R),Ovid MEDLINE(R) Daily Update,Ovid MEDLINE(R) In-Process,Ovid MEDLINE(R) Publisher; records were retained]	894
14	13 not 11	421
15	9 not 14	3050
16	(adult or adulthood or adults or centenarian* or elderly or geriatric* or "middle age" or "middle aged" or nonagenarian* or octogenarian* or "old adult*" or "old people" or "old person*" or "older adult*" or "older people" or "older person*" or septuagenarian* or Sextenarian* or "very old").ti,ab,hw,kw	16898479
17	(newborn* or neonat* or infant* or toddler* or child* or adolescent* or paediatric* or pediatric* or girl or girls or boy or boys or teen or teens or teenager* or preschooler* or "pre-schooler*" or preteen or preteens or "pre-teen" or "pre-teens" or youth or youths).ti,ab,hw,kw	8849248
18	17 not 16	5101406
19	15 not 18	2719
20	limit 19 to (editorial or erratum or note or addresses or autobiography or bibliography or biography or blogs or comment or dictionary or directory or interactive tutorial or interview or lectures or legal cases or legislation or news or newspaper article or overall or patient education handout or periodical index or portraits or published erratum or video-audio media or webcasts) [Limit not valid in CCTR,CDSR,Embase,Ovid MEDLINE(R),Ovid MEDLINE(R) Daily Update,Ovid MEDLINE(R) In-Process,Ovid MEDLINE(R) Publisher; records were retained]	115
21	19 not 20	2604
22	Remove duplicates from 21	1858

### Scopus

1. TITLE-ABS-KEY(((Duchenne or Becker) W/3 carrier*) OR "becker dystroph*" OR "becker muscular dystroph*" OR "becker syndrome*" OR "becker type muscular dystroph*" OR "beckers dystroph*" OR "beckers muscular dystroph*" OR "beckers syndrome*" OR "beckers type muscular dystroph*" OR "beckers-type progressive muscular dystroph*" OR "becker-type progressive muscular dystroph*" OR BMD OR "childhood pseudohypertrophic muscular dystroph*" OR DMD OR "duchenne dystroph*" OR "duchenne muscular dystroph*" OR "duchenne syndrome" OR "duchenne type muscular dystroph*" OR "duchennes dystroph*" OR "duchennes muscular dystroph*" OR "duchennes syndrome" OR "duchennes type muscular dystroph*" OR "duchennes-type progressive muscular dystroph*" OR "duchenne-type progressive muscular dystroph*" OR dystrophinopath* OR "morbus duchenne*" OR "pseudo hypertrophic myopathic progressive muscular dystroph*" OR "pseudohypertrophic childhood muscular dystroph*" OR "pseudohypertrophic muscular dystroph*" OR "X linked dilated cardiomyopath*" OR "XL-dCM")
2. TITLE-ABS-KEY(("glycogen storage disease*" W/5 (heart or cardiac* or myocard*)) or adhalinopath* or "alpha sarcoglycanopath*" or "alpha-sarcoglycanopath*" or "antopol disease*" or "arrhythmogenic right ventricular dysplasia" or "asymmetric septal hypertrophy" or "Barth syndrome" or cardiomyopath* or "cardioskeletal myopathy with neutropaenia and abnormal mitochondria" or "cardioskeletal myopathy with neutropenia and abnormal mitochondria" or "cardiovascular trypanosomiasis" or carditis or "chagas myocarditis" or "chronic progressive external ophthalmoplegia with myopath*" or "congestive heart disease*" or "cpeo with myopath*" or "cpeo with ragged red fiber*" or "danon disease*" or "diabetic cardiomyopath*" or "endocardial fibroelastos*" or "endomyocardial fibroelastos*" or "endomyocardial fibros*" or "familial ventricular hypertrophy" or "glycogen storage disease iib" or "glycogen storage disease type 2b" or "glycogen storage disease type iib" or "heart amyloidos*" or "heart myopath*" or "heart right ventricle dysplasia*" or "hereditary ventricular hypertroph*" or "isolated left ventricular noncompaction" or "isolated noncompaction of the ventricular myocardium" or "isolated non-compaction of the ventricular myocardium" or "isolated noncompaction of ventricular myocardium" or "isolated ventricular noncompaction" or "kearn sayre mitochondrial cytopath*" or "kearn syndrome" or "kearns sayre shy daroff syndrome" or "Kearns Sayre syndrome" or "kearns syndrome" or "kearn-sayre mitochondrial cytopath*" or "kearns-sayre mitochondrial cytopath*" or "kearns-sayre syndrome" or "kearns-sayre-shy-daroff syndrome" or "keshan disease*" or "left ventricular noncompaction" or "left ventricular non-compaction" or "lysosomal glycogen storage disease with normal acid maltase" or "lysosomal glycogen storage disease without acid maltase deficiency" or "muscular dystrophy limb girdle with alpha sarcoglycan deficiency" or "muscular dystrophy limb-girdle with alpha-sarcoglycan deficiency" or "myocardial disease*" or "myocardial ischemic reperfusion injur*" or "myocardial reperfusion injur*" or myocardiopath* or myocarditides or myocarditis or "noncompaction of the ventricular myocardium" or "noncompaction of ventricular myocardium" or "nonobstructive hypertrophic myocardiopath*" or "obstructive asymmetric septal hypertrophy" or "oculocraniosomatic syndrome*" or "ophthalmoplegia plus syndrome*" or "primary adhalinopath*" or "primary myocardial disease*" or "pseudoglycogenosis 2" or "pseudoglycogenosis 2 s" or "pseudoglycogenosis ii" or "pseudoglycogenosis iis" or sarcoglycanopat* or "secondary myocardial disease*" or "ventricular noncompaction" or "ventricular non-compaction" or "X linked cardioskeletal myopathy and neutropaenia" or "X linked cardioskeletal myopathy and neutropenia")
3. LANGUAGE(english or spanish)
4. 1 and 2 and 3
5. TITLE-ABS-KEY(newborn* or neonat* or infant* or toddler* or child* or adolescent* or paediatric* or pediatric* or girl or girls or boy or boys or teen or teens or teenager* or preschooler* or "pre-schooler*" or preteen or preteens or "pre-teen" or "pre-teens" or youth or youths) AND NOT TITLE-ABS-KEY(adult or adults or "middle age" or "middle aged" OR elderly OR geriatric* OR "old people" OR "old person*" OR "older people" OR "older person*" OR "very old")
6. 4 and not 5
7. DOCTYPE(ed) OR DOCTYPE(bk) OR DOCTYPE(er) OR DOCTYPE(no) OR DOCTYPE(sh)
8. 6 and not 7
9. INDEX(embase) OR INDEX(medline) OR PMID(0* OR 1* OR 2* OR 3* OR 4* OR 5* OR 6* OR 7* OR 8* OR 9*)
10. 8 and not 9
